# Multi-antigen print immunoassay (MAPIA)-based evaluation of novel recombinant *Leishmania infantum* antigens for the serodiagnosis of canine visceral leishmaniasis

**DOI:** 10.1186/s13071-015-0651-6

**Published:** 2015-01-24

**Authors:** Isaac Queiroz de Oliveira, Rodrigo Araujo Silva, Michel Vergne Sucupira, Edmilson Domingos da Silva, Alexandre Barbosa Reis, Gabriel Grimaldi, Deborah Bittencourt Mothé Fraga, Patrícia Sampaio Tavares Veras

**Affiliations:** Laboratório de Patologia e Biointervenção, Centro de Pesquisas Gonçalo Moniz, FIOCRUZ, Rua Waldemar Falcão, 121 (Candeal), Salvador, BA Brazil; Laboratório de Tecnologia Diagnóstica, Instituto de Tecnologia em Imunobiológicos, Bio-Manguinhos, FIOCRUZ, Rio de Janeiro, RJ Brazil; Instituto de Ciência e Tecnologia de Doenças Tropicais, INCT-DT, Salvador, BA Brazil; Laboratório de Imunopatologia, Núcleo de Pesquisas em Ciências Biológicas, Universidade Federal de Ouro Preto, Ouro Preto, MG Brazil; Departamento de Medicina Veterinária Preventiva e Produção Animal, Escola de Medicina Veterinária e Zootecnia, Universidade Federal da Bahia, Salvador, BA Brazil

**Keywords:** MAPIA, Recombinant antigens, Leishmaniasis

## Abstract

**Background:**

Domestic dogs are the principal reservoir hosts of *Leishmania infantum* in regions where visceral leishmaniasis (VL) is endemic. Although serologic methods are frequently used for the screening of infected dogs, antibody-based tests require further assessment, due to lack of sensitivity and specificity. In this study, we employed a multi-antigen printing immunoassay (MAPIA) to compare the antibody responses to novel recombinant proteins of *L. infantum* with the potential for the detection of canine VL.

**Findings:**

MAPIA strips were prepared employing 12 recombinant proteins. Antibody reactivity to these antigens was compared using a panel of sera collected from clinically asymptomatic (n = 16) and symptomatic (n = 41) culture-positive animals. Our findings showed that the canine immune response to antigen differs between dogs and depends on infection status. Using this screening assay, when five out of the 12 antigens were combined, an overall 81% detection rate of *L. infantum*-infected dogs was achieved.

**Conclusions:**

We conclude that MAPIA is an effective screening tool to rapidly select multiple antigens of diagnostic utility to be used in a more sensitive point of care diagnostic test such as the Dual-Path Platform (DPP) multiplex test for the rapid detection of infected dogs.

## Background

Zoonotic visceral leishmaniasis (VL) caused by *L. infantum* is an important emerging parasitic disease in many regions [1]. In the neotropics, *L. infantum* transmission to humans occurs as a result of *Lutzomyia longipalpis* bites [2]. Accordingly, dogs are the major source of *L. infantum* for humans; thus, early and accurate detection of infected dogs is critical to successfully controling the spread of leishmaniasis [3,4]. Additionally, it is also important to highlight the value of a reliable test to screen seronegative dogs before vaccination and to confirm infection before culling of seropositive dogs.

Current parasitological diagnostic tests, including microscopic examination and *in vitro* culturing, offer limited sensitivity with respect to the direct detection of *Leishmania*. In addition, parasite-specific antibody tests, such as the immunofluorescent-antibody test [IFAT], direct agglutination test [DAT], enzyme-linked immunosorbent assay [ELISA], although widely used to diagnose infection, employ crude antigens derived from whole-parasite extracts and lack the appropriate sensitivity and specificity required for accurate serodiagnosis [5-11].

Recently, the use of recombinant protein-based immunochromatographic testing, such as lateral-flow and Dual-Path Platform (DPP®) technologies, has overcome the practical limitations of other serological-based methods in the field [6,11-13]. Previous studies have indicated the promising potential of antigen-based serodiagnostic assays for VL, employing a cocktail of antigens [14] or chimeric proteins [15] that cover a broad spectrum of immunoreactivities [16-19]. Although these tests perform well, they do have limitations; for example, DPP® shows high sensitivity (98%) and specificity (96%) towards sera from symptomatic dogs, but shows a low sensitivity of only 47% towards sera from asymptomatic dogs [13].

With this in mind, we used the screening test, multi-antigen print immunoassay (MAPIA), to further characterize antibody responses in order to select those *L. infantum* recombinant proteins with a greater capacity to be utilized for the serodiagnosis of canine visceral leishmaniasis (CVL). MAPIA is more efficient, cost-effective, and reproducible than other screening techniques. In addition, as MAPIA is a membrane-based assay, it can easily be developed into a rapid test that utilizes thin-layer immunochromatography, similar to rapid diagnostic tests for other infectious diseases [20]. This advantage is important because our future goal is to generate a more reliable DPP® assay [13], using MAPIA to carefully select multiple antigens for the effective serodiagnosis of *L. infantum*-infected dogs.

## Methods

### *Leishmania infantum* antigens

A set of 12 recombinant *L. infantum* antigens (rLci1A, rLci2B, rLci3, rLci4, rLci5, rLci6, rLci7, rLci8, rLci10, rLci11, rLci12, rLci13) was previously selected from DNA libraries based on antibody reactivity using sera from culture-positive dogs [21,22]. Histidine-tagged recombinant proteins were produced after sub-cloning DNA fragments as described previously [21]. The antigens were then purified by affinity chromatography using PD-10 Desalting Workmate nickel-sepharose columns (Amersham Pharmacia Biotech AB, Sweden), in accordance with the manufacturer’s instructions.

### Dog sera and infection status

A panel of 138 canine sera was used. Negative control sera were obtained from 40 kennel dogs from Pelotas, Rio Grande do Sul (a VL-free area of Brazil). These dogs tested negative for *L. infantum* via serology, culturing, and qPCR of splenic aspirate [23]. To test for cross-reactivity of the 12 recombinant antigens with other pathogens, we also screened sera from dogs infected with *Leishmania braziliensis* (n = 10), *Trypanosoma cruzi* (n = 10), *Babesia* spp. (n = 10), and *Ehrlichia canis* (n = 11). To determine sensitivity, the antibody reactivity was assessed using a panel of 57 sera from symptomatic (n = 41) and asymptomatic (n = 16) culture-positive dogs. All infected dogs enrolled in the study were selected during epidemiological surveys of CVL carried out in four endemic areas in Brazil: Camaçari, Bahia; Dias D’Àvila, Bahia; Jequié, Bahia; and Pancas, Espírito Santo. At the time of sampling, dogs were clinically examined for seven typical signs of CVL and were scored clinically as asymptomatic if they had total scores of 0 to 4 and as symptomatic if they had scores greater than 4 [8].

### MAPIA strip preparation

Antigens were sprayed onto a 0.45-μm, pore-size nitrocellulose membrane (HiFlow Plus HFB24004, Millipore, MA) in parallel bands via use of a semi-automatic air-brush printing device (CAMAG automatic TLC sample 4, CAMAG, Muttenz, Switzerland) with a volume of 5 μL/mm. As described by Lyashchenko and collaborators [20], each antigen solution was printed in 15 cm length lines using the concentration of antigen according to solubility in phosphate-buffered saline (PBS): Lci1 = 0.236 mg/mL, Lci2 = 0.222 mg/mL, Lci3 = 0.530 mg/mL, Lci4 = 0.055 mg/mL, Lci5 = 0.139 mg/mL, Lci6 = 0.347 mg/mL, Lci7 = 0.097 mg/mL, Lci8 = 0.125 mg/mL, Lci10 = 0.139 mg/mL, Lci11 = 0.055 mg/mL, Lci12 = 0.236 mg/mL, Lci13 = 0.180 mg/mL. Three additional lines were saturated with *L. major* lysate = 0.7620 mg/mL, recombinant CRA&FRA *T. cruzi* proteins = 0.290 mg/mL, and a protein A solution = 0.200 mg/mL. The printed nitrocellulose membranes were dried in ambient air and cut into 5-mm strips.

### Serum incubation and antibody detection

Before incubation with test sera, strips were blocked for 1 h in 800 μl of PBS with 0.3% Tween 20 (Calbiochem, La Jolla, CA) and 5% instant nonfat dry milk at room temperature while rocking. Then, the strips were incubated with 1:100 dilution of each serum for 30 min at room temperature while rocking. After being triple-washed with PBS-Tween under agitation for 5 min at 37°C, the strips were incubated with 1 mL of goat anti-dog IgG antibodies conjugated with peroxidase (1:150 dilution), at room temperature for 1 h, and then washed twice. Enzyme activity was visualized by incubating the strips for 5 min with 1 mL of substrate-chromogenic solution (5 mg of DAB in 19,995 μL of PBS, with 5 μL of hydrogen peroxidase). To stop the reaction, the strips were rinsed extensively in distilled water at room temperature. The strips were then dried and immediately stored in dark conditions until the results were visually read and digitalized.

### Data analysis

Test positivity was evaluated by interpretation of serum antibody reactivity by visual detection of a brown band on the antigen-impregnated nitrocellulose strips. Epi Info 7 (CDC, Atlanta, Georgia - USA) was used to perform combinatorial analysis.

### Ethical approval for animal use

All experiments involving animals were performed in compliance with Brazilian federal law for animal experimentation (Law 11794). In conformity with the Oswaldo Cruz Foundation (FIOCRUZ) animal experimentation guidelines, and according to instructions outlined in the Brazilian Ministry of Health’s manual for the surveillance and control of VL. The present study was approved by the Institutional Review Board (CEUA protocol no. 015/2009) of the Gonçalo Moniz Research Center in Bahia, Brazil (CPqGM-FIOCRUZ/BA).

## Finding

**Figure 1:**
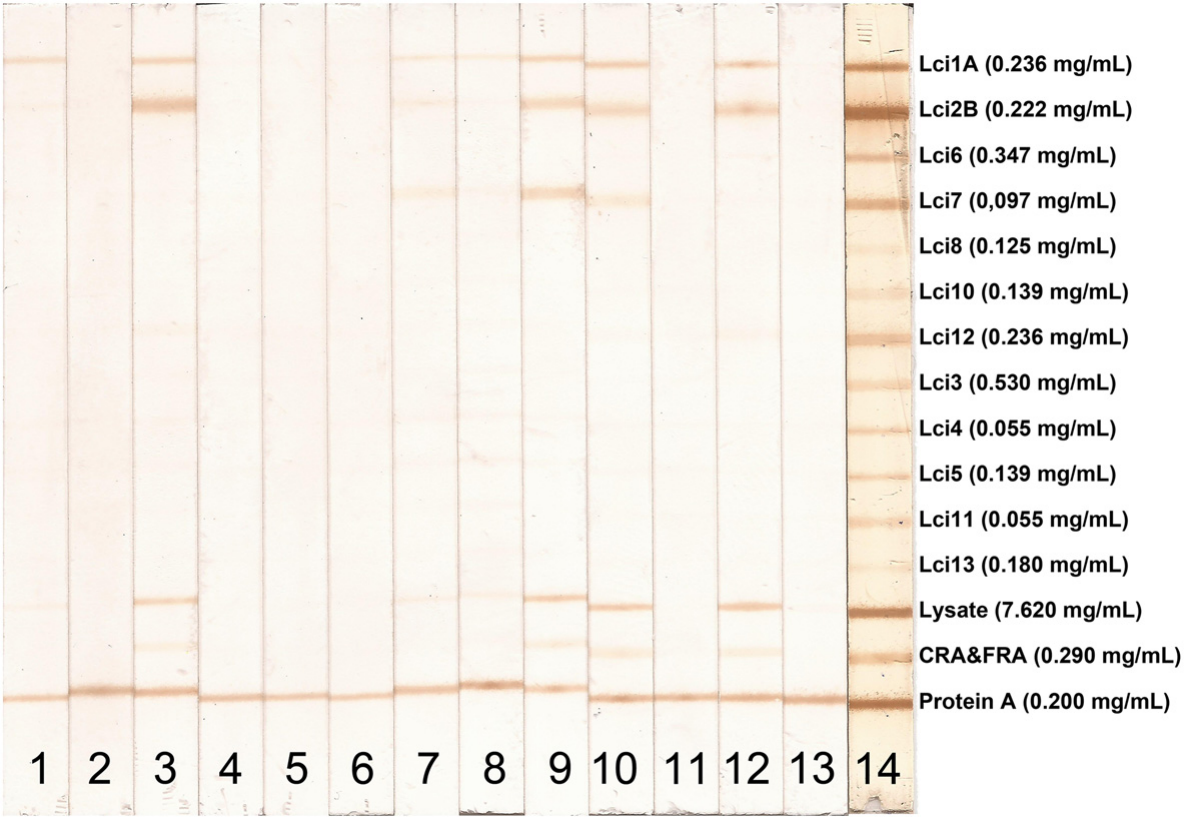
**MAPIA with recombinant** ***Leishmania infantum*** **antigens.** Images of strips containing printed individual antigens. Strips were incubated with serum samples from *L. infantum*-positive dogs (lanes 1, 3–13), serum from a normal control (uninfected) dog (lane 2), and the positive standard control serum pool (lane 14). The optimum concentration of each antigen wereLci1 = 0.236 mg/mL, Lci2 = 0.222 mg/mL, Lci3 = 0.530 mg/mL, Lci4 = 0.055 mg/mL, Lci5 = 0.139 mg/mL, Lci6 = 0.347 mg/mL, Lci7 = 0.097 mg/mL, Lci8 = 0.125 mg/mL, Lci10 = 0.139 mg/mL, Lci11 = 0.055 mg/mL, Lci12 = 0.236 mg/mL, Lci13 = 0.180 mg/mL, *L. major* lysate = 0.7620 mg/mL, recombinant CRA&FRA *T. cruzi* proteins = 0.290 mg/mL and protein A solution = 0.200 mg/mL. Antibody reactivity was detected as described in Methods. The test bands were visually read by two independent operators. Any visible band in the test area (in addition to the control line) was considered a positive reaction.

**Figure 2:**
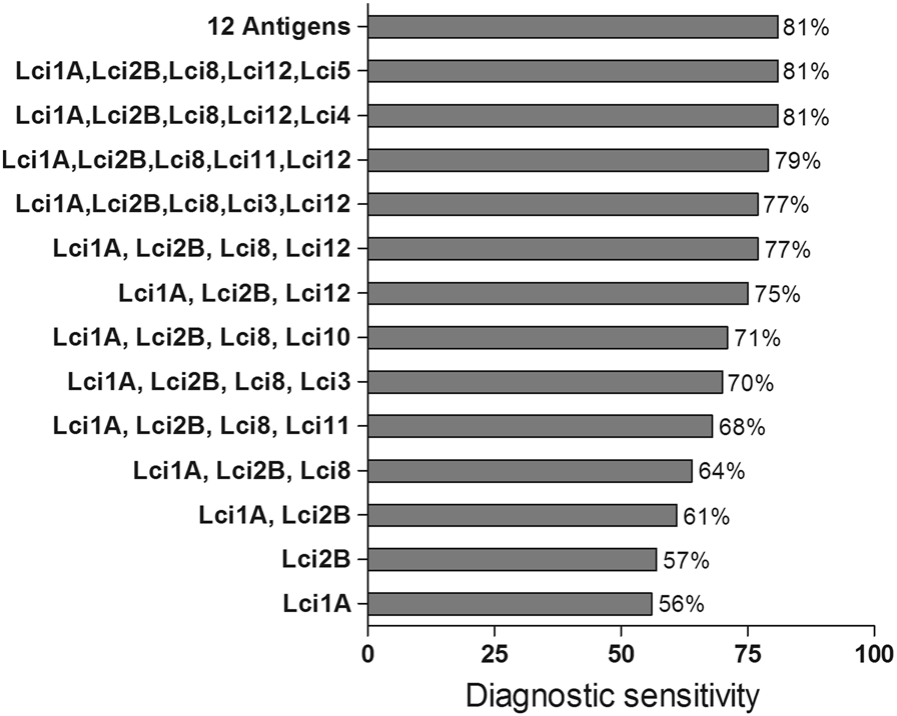
**Increased diagnostic sensitivity by combining the individual recombinant antigens for detecting clinically symptomatic and asymptomatic** ***Leishmania infantum*** **infected dogs.**

All MAPIA procedures were optimized with regard to antigen concentrations and serum dilution (data not shown). A total of 138 sera from clinically symptomatic (n = 41) and asymptomatic (n = 16) *L. infantum*-infected dogs, healthy controls (n = 40) and animals harboring other infections (n = 40) were tested against the selected panel of 12 antigens. The results showed variable antigen recognition patterns among the evaluated serum samples, as indicated in Figure 1. As shown in Table 1, the individual sensitivities of the 12 recombinant proteins coated onto nitrocellulose membranes ranged from 4 to 58% for identifying parasite-positive dogs. Nonetheless, each of the antigens detected some positive sera that others missed. When the individual recombinant proteins were combined, the total sensitivity increased to 81% (Figure 2), revealing that the antigens complemented each other. The well-known heterogeneous humoral immune response that develops in *L. infantum*-infected dogs [24] likely involves multiple antigens that are differentially recognized by the serum of each animal depending on the state of disease [8]. Therefore, further research into the development of a more reliable rapid test based on the combination of multiple antigens in a DPP format should be pursued.

**Table 1:** **Sensitivity and specificity of MAPIA with recombinant antigens of** ***Leishmania infantum*** **for the serodiagnosis of canine visceral leishmaniasis**

**Study groups (n)**	**Reactivity with individual** ***L. infantum*** **antigens (number of positive sera)**												
	**Lci1A**	**Lci2B**	**Lci3**	**Lci4**	**Lci5**	**Lci6**	**Lci7**	**Lci8**	**Lci10**	**Lci11**	**Lci12**	**Lci13**	**Any antigen**
Dogs without signs of VL (16)	3	2	4	6	4	1	2	1	4	2	6	1	9
Dogs with signs of VL (41)	29	31	8	15	15	1	9	4	3	8	13	3	37
Total (57)	32	33	12	21	19	2	11	5	7	10	19	4	46
% Sensitivity	56	58	21	37	33	4	19	9	12	18	33	7	81
Normal control (40)	0	1	2	2	2	1	3	1	2	2	4	3	6
Dermal leishmaniasis* (10)	6	3	0	2	2	0	1	0	0	0	0	0	6
Trypanosomiasis* (10)	1	1	1	1	1	0	1	0	1	0	3	0	3
Babesiosis* (10)	1	2	3	3	3	0	1	0	3	1	2	2	4
Ehrlichiosis* (11)	0	0	0	1	1	0	1	0	1	0	1	1	2
Total (81)	8	7	7	11	9	1	7	1	7	3	10	6	21
% Specificity	90	91	91	86	89	99	91	99	91	96	88	93	74

*Sera of dogs affected with other infections.

Efforts to develop a diagnostic test based on antibody-detection for VL or CVL have been underway for decades although few of such tests are chromatographic immunoassays currently used in endemic countries [12,13,25]. Prior studies have demonstrated that antibodies against these antigens are mainly detectable in cases with advanced stages of disease, while they are much less detectable in sera from asymptomatic cases [12,13]. Variable multi-antigen recognition by canine serum provides an explanation for the variable performance of commercial tests for detecting infected dogs. Here, we found the recombinant antigens evaluated were more sensitive in symptomatic cases (89%) in comparison to dogs without signs of VL (56%), thus confirming that these antigens are differentially recognized at different stages of infection. Using MAPIA, the Lci2B and Lci1A antigens gave the best results in terms of sensitivity (76% and 71%, respectively) for the detection of dogs with active disease. Although individually, none of these antigens performed well in detecting asymptomatic dogs (sensitivity ranging from 6 to 38%), 46 out of 57 serum samples recognized two groups of combined antigens (namely, rLci1A, rLci2B, rLci8, rLci12 and rLci4 or rLci1A, rLci2B, rLci8, rLci12 and rLci5), thus improving the overall sensitivity of the test to 81%. This result is in agreement with previously published results suggesting that more than one recombinant antigen may be useful to maximize the sensitivity of diagnostic tests for CVL [22,26].

As shown in Table 1, the individual antigens displayed variable specificities (ranging from 86% to 99%). The rLci1A antigen had a specificity of 90% and the rLci2B antigen had a specificity of 91%. These results are in accordance with previous study, which reported specificity values of 92% for rLci1A and 95% for rLci2B [27]. In addition, rLci8, which composes the groups of combined antigens, also showed a very high specificity value (99%). By contrast, the other three antigens, rLci4, rLci12 and rLci5, included in at least one of the two groups of combined antigens, showed lower specificity values of, respectively, 86, 88 and 89%, reducing overall specificity value to 74% (Table 1).

MAPIA has previously proven to facilitate rapid screening of antigens, since in comparison to ELISA [20], it is an easy and rapid test that simultaneously detects the response of sera to multiple antigens coated onto a single nitrocellulose strip. Indeed, the serological performance of antigens in MAPIA shown to be a good predictor of their performance in several point-of-care assays [14,15,28]. Here, we reveal MAPIA to be a useful tool for *L. infantum* antigen selection for the future development of an immunodiagnostic test for CVL. This screening test allowed the selection of two sets of antigens (rLci1A, rLci2B, rLci8, rLci12 and rLci4 or rLci1A, rLci2B, rLci8, rLci12 and rLci5) offering both high sensitivity and specificity; our results illustrate the benefit of utilizing a more effective multi-antigen point-of-care test in DPP format for application in mass assessment surveys of *L. infantum*-exposed dogs.
